# How Valid are Web-Based Self-Reports of Weight?

**DOI:** 10.2196/jmir.2393

**Published:** 2013-04-09

**Authors:** Stephanie Erika Bonn, Ylva Trolle Lagerros, Katarina Bälter

**Affiliations:** ^1^Department of Medical Epidemiology and BiostatisticsKarolinska InstitutetStockholmSweden; ^2^Clinical Epidemiology UnitDepartment of MedicineKarolinska InstitutetStockholmSweden

**Keywords:** body weight, Internet, validity

## Abstract

**Background:**

Many studies rely on self-reported anthropometric data. While paper-based self-reports have been the standard collection mode, the number of studies collecting self-reported data via the Web is increasing rapidly. Although numerous studies have shown good agreement between self-reported and measured weight using paper-based questionnaires, the validity of using the Web to inquire about weight is unknown.

**Objective:**

The objective of this study was to validate Web-based self-reports of bodyweight compared to weight measured at the study center.

**Methods:**

The validity of weight self-reported via the Web was assessed by comparing self-reports against measurements of weight in a convenience sample of 149 individuals (77.2% women, 115/149), aged 20-65 years. Study participants self-reported their weight via a Web-based questionnaire and thereafter had their weight measured in the research center.

**Results:**

The Spearman correlation coefficient between self-reported and measured weight was 0.98 (*P*<.001). The mean difference between self-reported and measured weight was -1.2 (SD 2.6) kg. There was a statistically significant difference between self-reported and measured weight with the self-reported being lower (*P*<.001). Subjects with a body mass index (BMI) ≥25 kg/m^2^, and subjects ≥30 years of age, under-reported their weight statistically significantly more than subjects with a BMI <25 kg/m^2^, and subjects <30 years of age, respectively.

**Conclusions:**

Our results show that self-reported weight via the Web can be a valid method of data collection.

## Introduction

Many studies rely on self-reported data on anthropometric variables. This is more practical and less expensive to collect, than measured data, especially when handling a large sample size. Nonetheless, self-reported data is limited by information bias [1]. Considering the increasing number of studies collecting Web-based data, there is a need to determine the validity of Web-based self-reports of body weight.

It has been suggested that using the Internet creates a distance between the investigator and the subject, as a self-reported questionnaire increases the anonymity of the subject responding. This makes it easier for the subject to answer personal questions truthfully and encourages reporting of uncensored personal information [2]. Sensitive or stigmatized behaviors have been reported more truthfully in self-administered surveys compared to interview-conducted surveys [3,4]. A previous study investigating the validity of self-reported weight by telephone interview and a paper questionnaire found that overweight and obese women reported weight more accurately in the questionnaire [5]. Heavy drinking of alcohol is another example of a stigmatized behavior where a Web-based questionnaire captured more heavy drinkers compared to telephone interviews [6].

While many studies have evaluated paper-based self-reports of weight, it is unclear how valid Web-based self-reports are. The aim of this study was to validate Web-based self-reports of weight against weight measurements made by the study personnel at a research center.

## Methods

### Study Design

Individuals between the ages of 20 and 65 years were recruited for participation during the spring of 2009 by public advertisements in Stockholm County, Sweden. The primary aim of the study was to validate methods assessing diet and physical activity. Access to the Internet and an email address were the requirements for participation. Subjects who were on any form of weight alteration diet, pregnant, or had given birth during the 10 months prior to the start of the study were excluded from participation. Participants were provided with information about the study and gave their written informed consent prior to the start of the study. The study design was previously described in detail by our research group [7]. The study was approved by the research ethics committee at Karolinska Institutet, Stockholm, Sweden.

In total, 179 subjects were included for participation in the study. Out of them, 150 subjects applied via the Web by filling in a questionnaire with personal information, including height and current weight. The questionnaire was downloaded from the Web, filled out by applicants on the computer, and thereafter sent via email to the researchers. There were no instructions regarding clothing or of subjects having to weigh themselves specifically for the questionnaire. Subjects were not aware that their weight was going to be measured at the start of the study when applying for participation. The remaining 29 subjects applied via personal contact or regular paper mail and were not included in the analysis of the present study. Approximately 2 months after applying for participation, subjects attended an introductory meeting and had their weight measured by study personnel. Subjects wore indoor clothing and no shoes during weight measurements. Measurements were made using a digital scale displaying weight to a tenth of a kg. All measurements were made by the same study personnel using the same scale. One subject out of the 150 who applied via the Web had no data on measured weight at the study center and was excluded from further analyses. Thus, 149 subjects were included in statistical analyses.

### Statistical Methods

Characteristics of the study participants are presented as descriptive statistics. Results of self-reported weight and height, measured weight, and differences between assessments, are reported as mean values and standard deviations. Potential differences between men and women, subjects <30 and ≥30 years of age, and subjects with a body mass index (BMI) <25 and ≥25 kg/m^2^, were assessed using paired *t* tests for continuous variables and chi-square tests for categorical variables. We also performed multivariate linear regression controlling for sex, age (<30 or ≥30 years), BMI (<25 or ≥25 kg/m^2^), education, and smoking. The degree of association between self-reported and measured weight was assessed using Spearman and intraclass correlation coefficients [8]. Because systematic differences between assessments cannot be detected using Spearman correlation coefficients, absolute agreement between the assessments was determined by using the Bland-Altman technique [9]. The difference between self-reported and measured weight (y-axis) was plotted against the mean of the two assessments (x-axis). The significance level was set to *P*<.05. All analyses were performed using STATA 11.1

## Results

Characteristics of subjects included in analyses are presented in Table 1. The majority of subjects were female (77.2%, 115/149), <30 years of age (51.7%, 77/149), and had a BMI <25 kg/m^2^ (76.5%, 114/149). Men reported using Swedish snuff, a moist form of snuff, to a higher extent than women (**P*=.*000). No other statistically significant differences were found between men and women, or between subjects with a BMI <25 or ≥25 kg/m^2^.

**Figure 1:**
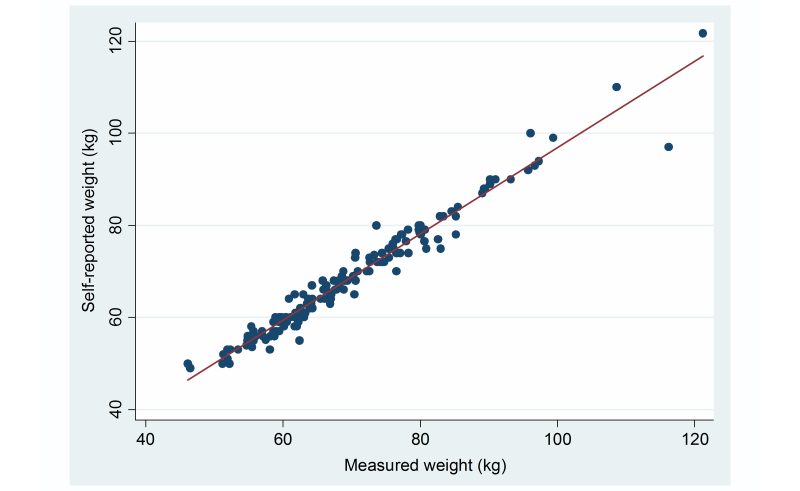
Scatter plot showing the correlation between self-reported (y-axis) and measured (x-axis) weight (kg). The Spearman correlation coefficient was 0.98 (*P*<.001). Each data point represents one subject. n=149.

The Spearman correlation coefficient between self-reported weight via the Web and weight measured by study personnel was 0.98 (**P*<.*001) for all subjects (Figure 1). The Spearman correlation coefficients for men and women were 0.89 (**P*<.*001) and 0.97 (**P*<.*001) respectively. For subjects with a BMI <25 kg/m^2^ and ≥25 kg/m^2^, the Spearman correlation coefficients between self-reported and measured weight were 0.97 (**P*<.*001) and 0.98 (**P*<.*001) respectively. Intraclass correlation coefficients were almost identical to the Spearman correlation coefficients (results not shown). There was a statistically significant difference between self-reported and measured weight in the whole group (*P*<.001), and in women (*P*<.001). There was a borderline statistical difference in men (*P*=.066). Further, there were statistically significant differences between self-reported and measured weight independent of BMI (<25 kg/m^2^, *P*<.001 and ≥25 kg/m^2^, *P*=.002).

**Figure 2:**
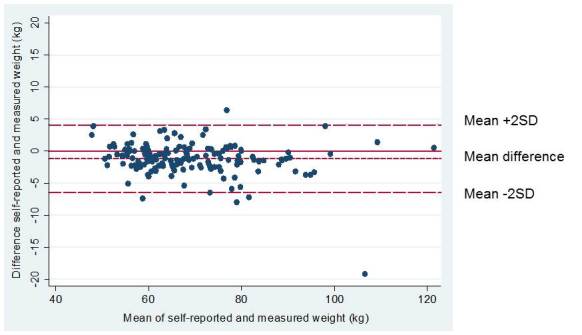
Bland-Altman plot showing the difference between self-reported and measured weight (y-axis) in relation to measured weights (kg). The mean difference was -1.2 kg. The 95% limits of agreement for the observations are represented as ±2 SD of the mean difference. Each data point represents one subject. n=149.

Figure 2 shows a Bland-Altman plot graphically illustrating differences between self-reported and measured weight among all subjects, the mean difference between self-reported and measured weight was -1.2 kg (SD 2.64). The plot shows good agreement between the two methods. However, the trend in the plot indicates increased under-reporting of self-reported weight with increasing body weight.

Table 2 shows self-reported weight, height and BMI, measured weight, and differences between self-reported and measured weight. Self-reported weight was under-reported compared to measured weight for all subjects (**P*<.*001). The difference between assessments remained highly significant among women, -1.3 kg (**P*<.*001), but not among men, -0.9 kg (*P*=.07). The difference between self-reported and measured weight was not statistically significantly different between men and women. For subjects with a BMI <25 kg/m^2^ and ≥25 kg/m^2^, there was a statistically significant difference between the groups (*P*=.02), with heavier subjects under-reporting on average -1.2 kg more compared to leaner subjects. Years of education did not appear to affect self-reports of weight while subjects ≥30 years of age under-reported their weight more than subjects <30 years of age (*P*=.02).

Results from the multivariate regression controlling for sex, age (<30 or ≥30 years), BMI (<25 or ≥25 kg/m^2^), education, and smoking were similar to the results described above. There were statistically significantly higher under-reporting among subjects ≥30 years and subjects with a BMI ≥25 kg/m^2^ compared to subjects <30 years of age and a BMI of <25 kg/m^2^, respectively. Sex, education, and smoking did not affect the difference between self-reported and measured weight (data not shown).

**Table 1:** Characteristics of study participants.

		All		Men		Women		*P* value^a^
		n=149		n=34		n=115		
		n	%	n	%	n	%	
**Age (years)**								.45
	<30	77	51.7	18	52.9	59	51.3	
	30-39	28	18.8	7	20.6	21	18.3	
	40-49	22	14.8	7	20.6	15	13.0	
	50-59	18	12.1	2	5.9	16	13.9	
	>60	4	2.7	0	0.0	4	3.5	
**Education (years)**								.46
	9-12^b^	32	21.5	9	26.5	23	20.0	
	>12	114	76.5	25	73.5	89	77.4	
	Missing data	3	2.0	0	0.0	3	2.6	
**Smoker**								.12
	Current	11	7.4	5	14.7	6	5.2	
	Previous	38	25.5	6	17.6	32	27.8	
	Never	96	64.4	23	67.6	73	63.5	
	Missing data	4	2.7	0	0.0	4	3.5	
**Swedish snuff^c^ user**								.000
	Current	7	4.7	6	17.6	1	0.9	
	Previous	16	10.7	7	20.6	9	7.8	
	Never	122	81.9	21	61.8	101	87.8	
	Missing data	4	2.7	0	0.0	4	3.5	
**BMI (kg/m^2^)^d^**								.11
	<25	114	76.5	27	79.4	87	75.7	
	≥25	35	23.5	7	20.6	28	24.3	

^a^comparing men and women using chi-square tests

^b^primary school

^c^a moist form of snuff

^d^BMI based on self-reported weight and height

**Table 2:** Weight, height, and BMI based on self-reported and measured data.

			Self-report Mean (SD)			Measured Mean (SD)	Difference^a^ Mean (SD)
		n	Weight (kg)	Height (cm)	BMI (kg/m^2^)	Weight (kg)	Weight (kg)
**All**							
		149	68.7 (12.6)	170.6 (9.5)	23.6 (3.8)	69.9 (13.2)	-1.2 (2.6)
**Gender**							
	Men	34	80.0 (7.8)	183.2 (7.3)	23.9 (2.2)	80.9 (8.5)	-0.9 (2.7)
	Women	115	65.3 (11.7)	166.9 (6.4)	23.5 (4.1)	66.6 (12.5)	-1.3 (2.6)
	*P* value		<.001	<.001	.55	<.001	.44
**BMI (kg/m^2^)**							
	<25	114	64.9 (9.7)	171.4 (9.6)	22.0 (1.7)	65.8 (10.0)	-0.9 (2.1)
	≥25	35	80.8 (13.5)	168.3 (8.8)	28.5 (4.3)	83.0 (13.9)	-2.1 (3.8)
	*P* value		<.001	.09	<.001	<.001	.02
**Age (years)**							
	<30	77	66.6 (10.8)	171.7 (9.7)	22.5 (2.6)	67.3 (11.5)	-0.7 (2.5)
	≥30	72	70.9 (14.0)	169.5 (9.2)	24.7 (4.4)	72.6 (14.3)	-1.7 (2.7)
	*P* value		.04	.14	<.001	0.01	.03

^a^difference between measured and self-reported weight

## Discussion

### General Discussion

Results from the present study show that Web-based self-reports of weight are highly correlated, although somewhat under-reported, with measured weight in a Swedish population. Although there were small differences between subgroups, overweight and obese subjects with a BMI ≥25 kg/m^2^ under-reported their weight on average by 1.2 kg more than normal weight subjects. Further, we found that subjects ≥30 years of age, under-reported their weight on average by 1.0 kg more than younger subjects. Under-reporting was associated with both increasing BMI and age independently. No differences were seen between men and women. To the best of our knowledge, the present study is among the first to examine the validity of Web-based self-reports of weight.

Our results are in line with previous studies validating paper-based self-reports of weight against measurements of weight, which have shown high correlations and high validity [5,10-14]. Nevertheless, self-reported weight is under-estimated in most populations [15], and by most individuals [5,12,16], although under-weight subjects have been seen to over-report weight [5]. Consistent with the results of previous studies where increasing body mass was associated with increased under-reporting [15,17,18], our results demonstrated increased under-reporting among subjects with a high BMI.

As was also shown in a previous study [19], we saw an indication of gender difference, with women under-reporting their weight to a greater degree than men. The lack of statistical significance between the sexes’ degree of under-reporting might be due to our small sample size and fewer participating men. The greater under-reporting detected among older subjects may partly be explained by the positive association between BMI and age and the increasing under-reporting seen with increasing BMI. Increased under-reporting in older subjects could also partly be explained by memory bias due to older subjects not keeping track of their current weight to the same extent as younger subjects. Previous studies have also shown age to have an effect on under-reporting [15].

The observed under-reporting of self-reported weight compared to measured weight, may partly be explained by true differences between the time of self-reported and measured weight. However, the time period between the self-report and the measurement was fairly short and participants were healthy and not on any weight changing regimen. Thus, we believe that large changes in weight during this period were unlikely to have occurred, and does not fully explain the difference. Nonetheless, a narrower time period between the self-reported and measured weights would be ideal to avoid fluctuations in weight over time. Some of the under-estimation might be explained by subjects self-reporting their weight without clothes, while the measurements of weight were made while wearing clothing. Although this may explain part of the under-estimation, it is not likely to have caused the entire difference. Another explanation for differences might be the fact that subjects self-reported their weight in different manners as no specific instructions for self-reports were given, causing random errors. Errors in the measurement of weight were unlikely, given that the same scale was used by the study personnel for all measurements.

Furthermore, the lack of measured height was also a limitation to the current study, and may have had an effect on estimates of BMI. However, previous studies have shown good validity of categorization according to BMI assessed from self-reported data compared to measured data [5]. Therefore, we believe that our estimates of BMI assessed from self-reported weight and height were fairly accurate.

The use of Web-based self-reports of data has numerous advantages compared to collecting data using paper-based questionnaires. Data quality can be improved by implementing automatic controls for missing data and answers out of a reasonable range. The quality of anthropometric data, with regard to missing and plausible answers, collected using a Web-based questionnaire, has been shown to be equal to, or better than, that of data collected using a paper version of the questionnaire [20]. While Web-based questionnaires may be superior to their paper-based predecessors when it comes to completeness of data, concerns have been raised regarding response rates and selection bias introduced by using online surveys [21]. We do not believe, however, that these concerns will be a problem when collecting data in future studies. Access to, and use of, the Internet in Sweden has increased considerably during the last decade with more than 90% of the adult population having access today [22].

Like many other convenience samples, the present study population comprised of men and women of different ages, but the majority of subjects were nonetheless young females residing in Stockholm County, limiting the generalizability of the results. A truly population-based study would improve generalizability, but was clearly unfeasible for practical reasons. In addition, the study subjects who applied for participation in the study knew that the aim of the study was to evaluate methods assessing diet and physical activity. They may therefore be more health conscious and motivated to participate than the average population, and thus prone to self-reporting their weight in a more truthful manner. Nonetheless, the study participants were not initially aware of the comparisons between self-reported and measured weight.

The self-selection of participants might have created a sampling bias yielding a stronger correlation and a decreased difference between assessment methods than in the general population. Of note is that the study subjects had a mean BMI comparable to the general Swedish population [23]. Nonetheless, future studies should focus on validity of self-reports by other sub-groups, such as elderly or obese. Despite these potential limitations, our results may be helpful when using self-reported weights in studies in young and normal weight populations.

### Conclusions

We have found Web-based self-reports of weight to be as good as paper-based self-reports of weight found in previous studies. Although more validation studies may be needed, our results showed that self-reported weight via the Internet is a suitable method of data collection for use in research and clinical work.

## Abbreviations

BMI: body mass index
